# TimesNet-BFT: Mitigating Network State Uncertainty in Byzantine Consensus via Deep Temporal Modeling

**DOI:** 10.3390/e28030302

**Published:** 2026-03-08

**Authors:** Haolong Wang, Haijun Liu, Yahui Liu, Hongliang Ma, Pan Gao

**Affiliations:** School of Information Science and Technology, Shihezi University, Shihezi 832000, China

**Keywords:** Byzantine fault tolerance, deep temporal modeling, network latency prediction, dynamic leader rotation, adaptive timeout, epistemic uncertainty

## Abstract

Byzantine fault tolerance (BFT) protocols serve as the cornerstone of data consistency in permissioned blockchains; however, their scalability is inherently constrained by stochastic leader-centric bottlenecks and rigid, non-adaptive timeout mechanisms. Existing rule-based heuristics often fail to capture high-entropy and time-varying network latency, leading to frequent view changes and severe performance degradation under network volatility. To mitigate this epistemic uncertainty, this paper proposes TimesNet-BFT, a novel entropy-aware optimization framework. By leveraging TimesNet’s transformation of one-dimensional time series into two-dimensional tensors for multi-periodicity analysis, the framework accurately characterizes stochastic nodal latency patterns to facilitate entropy-minimized dynamic leader election and adaptive timeout strategies. Extensive evaluations conducted on simulated and real-world trace-driven Internet of Vehicles (IoV) scenarios validate the proposed approach, achieving a prediction MAPE below 5% alongside robust zero-shot generalization. Notably, under high-entropy network conditions, the framework demonstrates up to a 191.9% increase in throughput and mitigates latency variance by 73.3%, effectively neutralizing the structural bottlenecks inherent to traditional information-agnostic protocols. Crucially, by mathematically decoupling consensus safety from AI prediction errors, the system introduces an aggressive liveness paradigm that maintains minimal control plane overhead while significantly enhancing the entropic stability of the consensus process.

## 1. Introduction

As distributed systems such as the Internet of Things (IoT) and Internet of Vehicles (IoV) continue to proliferate exponentially [1,2], blockchain technology faces unprecedented scalability challenges in its capacity as a core architecture for data consistency and security. Specifically, as empirically demonstrated in recent studies, IoV networks are resource-constrained environments severely limited by strict data transmission bottlenecks [3]. Consequently, reducing data payloads and optimizing communication efficiency are imperative to maintain the operational viability of vehicular systems. Among the spectrum of consensus mechanisms, Byzantine fault tolerance (BFT) protocols are extensively deployed in consortium and permissioned blockchains due to their deterministic finality and inherent fork-free nature [4,5]. However, classical BFT protocols, such as PBFT, suffer from quadratic communication complexity $O(n^{2})$, which severely inhibits their scalability in bandwidth-critical environments. Although optimized protocols like HotStuff [6] have reduced this complexity to linearity by adopting a star topology, their performance remains constrained by two intrinsic bottlenecks: the bandwidth constraints of the leader node and rigid timeout mechanisms that are agnostic to the stochastic nature of distributed environments. Traditional BFT protocols exhibit a significant reliance on a single leader for proposal ordering and message aggregation. Research indicates that in high-concurrency environments, the leader node frequently becomes the system’s principal straggler [4]. This phenomenon increases the system’s vulnerability to Distributed Denial of Service (DDoS) attacks targeting the leader, resulting in a drastic degradation in system throughput. Furthermore, existing approaches typically rely on fixed timeout thresholds to detect leader failures, yet such static configurations exhibit poor adaptability when deployed in heterogeneous networks characterized by high entropy and dynamic volatility, such as IoV or Wide Area Networks (WAN) [7]. An overly aggressive timeout interval may be triggered by stochastic network noise, inducing frequent and computationally expensive view changes due to misinterpreting benign latency as node failure. Conversely, an overly conservative timeout setting results in prolonged long-tail latency during failure recovery, thereby severely compromising system availability.

To mitigate these limitations and reduce uncertainty in consensus scheduling, recent literature has explored integrating Artificial Intelligence (AI) into the consensus layer to facilitate protocol self-adaptation. A predominant paradigm involves deep reinforcement learning. For instance, general frameworks have leveraged Reinforcement Learning (RL) for dynamic parameter tuning, while specific approaches like BFT-Brain [8] utilized proximal policy optimization to adapt to variable attack vectors. However, RL-based approaches are often limited by high sample complexity and slow convergence rates, rendering them ineffective for responding to millisecond-level fluctuations in network entropy. Alternatively, reputation models based on statistical heuristics [9] elect leaders via historical scoring. However, such approaches inherently struggle to capture the complex, time-varying dynamics of network latency—particularly periodic tidal patterns obscured by stochastic noise—thereby hindering fine-grained latency prediction.

Addressing these deficiencies, this paper proposes a novel BFT consensus optimization framework that integrates advanced time-series analysis models to minimize state uncertainty. Departing from basic classification or regression approaches, we incorporate the TimesNet model, introduced at ICLR 2023 [10]. As a foundational time-series model, TimesNet transforms 1D temporal sequences into 2D tensors by leveraging multi-scale 2D convolutions to disentangle meaningful multi-periodicity from stochastic noise. By harnessing the robust feature extraction capabilities of TimesNet, we design a proactive leader election mechanism and an adaptive timeout strategy. Specifically, by effectively predicting the response latency of nodes for the subsequent view, the system proactively promotes the node with the lowest predicted latency to leadership and dynamically constrains the timeout window, thereby aligning protocol execution with the predicted low-entropy states. The primary contributions of this paper are summarized as follows:

1. To the best of our knowledge, this work represents a novel integration of TimesNet within the blockchain consensus domain. By exploiting its multi-periodicity analysis capabilities, we effectively address the challenge of predicting network latency in non-linear, high-entropy environments, thereby circumventing the limitations inherent in traditional statistical methods.
2. We formulate synergistic optimization mechanisms, including a dynamic leader rotation strategy and an adaptive timeout algorithm based on predictive scoring. This dual approach achieves a simultaneous enhancement of consensus efficiency and stability by reducing the uncertainty associated with leader selection and timeout configuration.
3. We conducted multidimensional evaluations under controlled high-entropy scenarios. The results demonstrate that TimesNet effectively mitigates the phase delay inherent in Recurrent Neural Networks (RNNs), achieving seamless proactive switching. Furthermore, the model demonstrates practical feasibility by performing inference in a non-blocking background thread, ensuring minimal computational overhead in real-time consensus environments.

## 2. Related Work

### 2.1. Optimization Strategies for BFT Consensus

BFT mechanisms underpin data consistency in consortium blockchains and distributed ledger technology. However, the quadratic communication complexity $O(n^{2})$ inherent in the classical PBFT algorithm severely constrains its scalability within expansive networks. To circumvent this limitation, contemporary scholarship has mainly focused on three distinct trajectories: sharding techniques, topological optimization, and domain-specific customization.

Within the domain of sharding and parallel processing, researchers employ partitioning strategies to enhance system throughput. For instance, IShard [11] leverages jump-consistent hashing algorithms to optimize shard configuration, thereby successfully reducing communication complexity to O(n). Addressing the threat of adversarial entities, USMN-SCA [12] presents a sharding scheme capable of tolerating an unbounded number of malicious nodes by utilizing a two-phase consensus to guarantee cross-shard security. To balance the trade-off between privacy and computational efficiency, Duo-H [13] and DRDST [14] introduce dynamic sharding mechanisms for dual-layer consortium blockchains and Internet of Vehicles scenarios, respectively. Notably, the latter employs tree-structured broadcasting to significantly reduce consensus latency. Furthermore, FDSS [15] mitigates storage overhead by decoupling on-chain metadata from off-chain storage repositories.

In terms of topological innovation, Directed Acyclic Graphs (DAGs) have garnered significant attention attributable to their high concurrency capabilities. Specifically, DAG-D [16] utilizes the DAG structure of transaction sets to achieve substantial throughput gains in industrial IoT environments. Similarly, Zebra [17] and its derivatives introduce cluster-tree topologies, effectively alleviating the bandwidth bottleneck associated with single-leader architectures. To accommodate heterogeneous network environments, the PoAh algorithm [18] explores hybrid consensus mechanisms, aiming to balance decentralization and execution efficiency.

In the context of highly dynamic and resource-constrained scenarios such as IoV and Unmanned Aerial Vehicle (UAV) networks, traditional static consensus faces formidable challenges. Schemes such as UAV-State-Driven [19] introduce state functions and hybrid voting to accommodate rapid topological evolution. Meanwhile, Jamming-Resilient [20] specifically employs anti-interference mechanisms to preserve consensus liveness in adversarial wireless environments. On the security front, anticipating the emergence of quantum computing, works such as QBIoT [21] and Post-Quantum DPoL [22] have pioneered the integration of post-quantum signatures and quantum random number generators, thereby establishing a foundation for quantum-resistant consensus.

Despite these significant architectural and security advancements, existing protocols continue to depend on deterministic or semi-deterministic timeout mechanisms, such as fixed thresholds and static exponential backoff strategies. When confronted with the stochastic fluctuations of real-world networks characterized by tidal congestion in IoV or bursty jitter in wireless links, these rigid consensus mechanisms frequently suffer from spurious view changes or long-tail latency. This vulnerability stems from the lack of resilience to proactively perceive the epistemic uncertainty inherent in network dynamics.

### 2.2. Data-Driven Consensus Adaptation and Intelligent State Estimation

Driven by the proliferation of AI, researchers have increasingly sought to integrate AI paradigms into blockchain consensus mechanisms and network performance optimization. Contemporary scholarship primarily focuses on intelligent reputation assessment, hyperparameter optimization via RL, and security fortification.

Reputation-driven leader selection represents the predominant paradigm in current AI-consensus integration. To mitigate the stochastic inefficiencies inherent in random leader selection, protocols such as RE-PBFT [23] and RRCA [24] incorporate multidimensional reputation models. These approaches leverage decision trees such as ID3 or weighted stochastic algorithms to dynamically elect leaders based on historical behavioral metrics, including online availability and block generation efficiency, thereby enhancing system robustness. Similarly, the TP-PBFT framework [25] optimizes consensus group composition by quantifying nodal trust values, while the Louvain-Committee approach [26] employs community detection algorithms to refine cross-chain committee generation. In medical and Federated Learning (FL) contexts, the LPOC protocol [27] further integrates nodal contribution to model training into the consensus incentive mechanism, thereby effectively realizing a “Contribution-as-Stake” paradigm.

Regarding parameter adaptation and resource optimization, RL has been extensively deployed. CO-ETS [28] adopts meta-heuristic algorithms to optimize task scheduling and consensus participation for edge computing nodes. Furthermore, the Energy-Efficient Consensus protocol [29] synthesizes power grid monitoring data with AI-driven forecasting to optimize block generation intervals. Recent state-of-the-art frameworks such as Blockchain-MLTrustNet [30] further integrate deep RL with adaptive chained blockchains to significantly improve trust management and node evaluation within IoV networks.

Within the domain of AI-enabled security, GenAI-DAA [31] utilizes generative AI to impute incomplete data, thereby augmenting consensus robustness. AI-DTMS [32] combines homomorphic encryption with machine learning to detect malicious vectors during the consensus process while preserving privacy. Furthermore, recent explorations into AI Agents [33] and Meta-Governance [34] have begun employing autonomous agents to identify smart contract vulnerabilities and governance risks.

Despite the intelligence introduced by these methodologies, existing solutions exhibit critical limitations when deployed in dynamic, resource-constrained environments. From an information-theoretic and systems perspective, these deficiencies manifest in three primary dimensions.

First, regarding prediction granularity, the majority of reputation mechanisms [23,24], and RL agents fundamentally operate as coarse-grained state estimators over prolonged epochs. Consequently, they lack the capacity to quantify the continuous entropy of network latency variables, whereas optimal view change decisions and precise timeout calibration necessitate millisecond-level latency anticipation. Second, concerning time-series adaptability, existing statistical heuristics and standard sequential models (e.g., LSTM or Transformers) struggle to disentangle “Multi-scale Periodicity” from stochastic noise, such as diurnal load variations mixed with microsecond-level jitter. They often suffer from slow convergence when confronted with sudden concept drift. In contrast, transforming 1D latency sequences into 2D tensors allows for instant adaptation to structural entropy shifts without reactive retraining. Finally, in terms of computational overhead, the continuous state-action evaluations inherent in DRL frameworks and the quadratic attention complexity in Transformers introduce a significant computational burden. This overhead is prohibitive for real-time consensus critical paths, necessitating lightweight, asynchronous forward-pass operations. To bridge these gaps, TimesNet-BFT is proposed to provide fine-grained, entropy-aware temporal modeling with minimal overhead.

### 2.3. Deep Temporal Modeling for Non-Stationary Network Dynamics

To address the aforementioned theoretical gaps and minimize system uncertainty, this paper integrates TimesNet [10], a foundational general-purpose time-series model. In contrast to traditional architectures such as RNNs or 1D convolutions [35], TimesNet introduces the temporal 2D-variation mechanism. The model leverages the Fast Fourier Transform (FFT) to extract dominant frequencies within the temporal data, thereby transforming 1D latency sequences—often indistinguishable from high-entropy noise—into 2D tensors. This dimensional transformation enables the application of 2D convolutional kernels to simultaneously capture intra-period short-term fluctuations and inter-period long-term trends.

The integration of TimesNet into the BFT consensus layer marks a transition from rule-based passive adaptation to proactive entropy reduction driven by deep temporal representation. This capability enables the protocol to move beyond binary classification of node integrity, facilitating the precise regression of future latency. Consequently, this mechanism facilitates adaptive leader rotation and dynamic timeout regulation, effectively acting as an information filter that stabilizes the consensus process against environmental volatility.

## 3. Methodology

**Figure 1:**
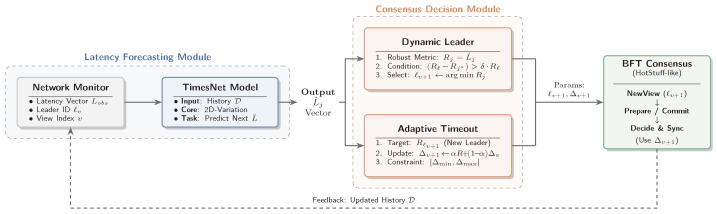
System Architecture of TimesNet-BFT. The Latency Forecasting Module extracts multi-period features from stochastic latency noise. These predictions are subsequently utilized by the Consensus Decision Module to map consensus views *v* (establishing $\ell_{v}\equiv \ell_{t}$) for dynamic leader election and adaptive timeout calibration.

This section presents TimesNet-BFT, a proposed entropy-aware optimization framework for BFT consensus underpinned by the TimesNet architecture. The core methodology encompasses two synergistic mechanisms: the utilization of TimesNet for multi-node latency forecasting to minimize state uncertainty for dynamic leader election, and the adaptive calibration of consensus timeout thresholds and rotation parameters based on these predictive priors. As illustrated in Figure 1, the architecture comprises two core modules: a latency forecasting module that acts as a temporal feature extractor, and a consensus decision module. Crucially, the predictions generated by the model serve solely as an auxiliary reference for consensus scheduling. The fundamental voting rules and commit logic remain strictly invariant. This design choice ensures that the protocol can gracefully degrade to the baseline consensus mechanism under any high-entropy anomalies, thereby guaranteeing the system’s safety.

### 3.1. Deep Temporal Modeling for Latency Uncertainty Reduction

We first formulate the latency prediction problem by establishing a formal representation of the input data, treating network latency as a non-stationary stochastic process. At the end of each consensus view *t*, the system records the historical latency metrics of all participating nodes. We formally define latency based on the role of the node in the current view. Specifically, for a non-leader node, the metric denotes the response duration ranging from receiving the proposal to emitting a vote message. Conversely, for the leader node, it captures the round-trip consensus duration extending from proposal dissemination to final commitment. Consequently, the leader’s latency $L_{\ell_{t}}\left(t\right)$ serves as a proxy for the total consensus latency of the view. The system records the leader identifier $\ell_{t}$, and the latency vector for the entire cluster of *N* nodes, denoted as ${\left\{L_{j}\left(t\right)\right\}}_{j=1}^{N}$. Accordingly, the aggregated observation set over a history of *T* views is formalized as:

(1) $$D={\left\{\left(\ell_{t},\;{\left\{L_{j}\left(t\right)\right\}}_{j=1}^{N}\right)\right\}}_{t=1}^{T}$$

For any node *i*, let $L_{i}\left(t\right)$ denote its observed latency in view *t*. The objective is to approximate a non-linear mapping function $f_{\theta }$, where θ represents the set of learnable model parameters. This function forecasts the latency of the target view by extracting deterministic patterns from stochastic history. Formally, we define the input vector $x_{i}\left(t\right)={\left[L_{i}\left(t-k\right),\ldots ,L_{i}\left(t-1\right)\right]}^{⊤}$ as the historical latency sequence of node *i* over a predefined lookback window of size *k*. The prediction is formulated as follows:

(2) $${\hat{L}}_{i}\left(t\right)=f_{\theta }\;\left(x_{i}\left(t\right)\right)$$

To capture the multi-scale periodic components hidden within the high-entropy latency sequences, we employ TimesNet [10]. In contrast to heuristic methods based on simple autocorrelation, TimesNet identifies the dominant periods by analyzing the series in the frequency domain via FFT. This process effectively acts as a spectral filter, separating the periodic signal from the stochastic noise. Specifically, the 1D sequence $x_{i}\left(t\right)$ is transformed into a 2D tensor based on the principal period $T_{i}$:

(3) $$X_{i}^{2D}\left(t\right)={\mathrm{Reshape}}_{\;T_{i}}\;\left(\mathrm{Padding}(x_{i}\left(t\right))\right)\in R^{F_{i}\times T_{i}}$$

where the Padding(·) operator extends the 1D temporal sequence with zeros at the terminus to ensure its total length is strictly divisible by the principal period $T_{i}$. The ${\mathrm{Reshape}}_{\;T_{i}}\left(\cdot \right)$ operator subsequently folds this padded 1D sequence into a 2D tensor of dimensions $F_{i}\times T_{i}$. Here, $F_{i}=\left\lceil k/T_{i}\right\rceil$ denotes the number of periods captured within the window *k*. This 2D representation allows 2D convolutional kernels to extract structured latency features, thereby reducing the epistemic uncertainty of the temporal model.

To address scenarios characterized by multiple latent periodicities, the TimesBlock mechanism within TimesNet adaptively characterizes multi-scale temporal structures by performing convolution and aggregation on the 2D temporal tensor $X_{i}\left(t\right)$ to yield the forecast ${\hat{L}}_{i}\left(t\right)$.

Regarding model configuration, we adopt a shared-weight architecture augmented with node-specific embeddings $e_{i}\in R^{d}$, where *d* is the embedding dimension encoding node identity. To mitigate the impact of stochastic outliers characterized by high aleatoric uncertainty, we employ a robust deep temporal regression mechanism. Instead of relying on simple linear fitting, our model optimizes for the conditional expectation of the latency distribution while explicitly suppressing error variance. We define this robust predictive metric as $R_{i}={\hat{L}}_{i}\left(t\right)$. During the offline training phase, the objective function minimizes the Mean Squared Error (MSE):

(4) $$L\left(\Theta ;S\right)=\frac{1}{\left|S\right|}\sum_{(i,t)\in S}{\left(L_{i}\left(t\right)-{\hat{L}}_{i}\left(t\right)\right)}^{2}$$

where Θ represents the model parameters, and S denotes the training sample set. Minimizing the MSE is theoretically equivalent to minimizing the variance of the prediction error $\mathrm{Var}(L-\hat{L})$. From an information-theoretic perspective, minimizing the variance of a distribution directly minimizes its differential entropy (as entropy scales logarithmically with variance $\sigma^{2}$). Consequently, this optimization compels the model to capture the true structural periodicity (low entropy) rather than fitting the stochastic noise (high entropy). Furthermore, we leverage transfer learning by initializing the model with pre-trained weights from standard time-series datasets provided by the TimesNet open-source repository. This strategy effectively accelerates convergence and enhances few-shot generalization capabilities in data-scarce scenarios.

In the subsequent online phase, to ensure long-term robustness against concept drift induced by structural network shifts while maintaining the determinism required for BFT safety, our framework incorporates a lazy recalibration strategy supported by fuzzy verification. In contrast to real-time backpropagation, which introduces non-deterministic floating-point risks, we maintain static model parameters for the consensus critical path. For long-term maintenance, we allow for periodic, asynchronous model weight updates via a dedicated governance view. To tolerate potential minor discrepancies between replica inferences due to hardware heterogeneity, we implement a tolerance threshold ϵ, detailed in the consensus decision logic (Section 3.2). This design ensures the model stays representative of current network conditions without sacrificing the strict safety invariants of the consensus process.

### 3.2. Proactive Leader Rotation Strategy for Entropy Minimization

Before detailing the adaptive leader rotation mechanism, we first formalize the trust model assumptions underpinning the AI-driven consensus layer. The framework operates under the standard Byzantine threat model in a partially synchronous network of N=3f+1 replicas, tolerating up to *f* nodes exhibiting arbitrary malicious behavior (including collusion and data falsification). Crucially, the deep temporal model and its predictive outputs are strictly treated as an untrusted oracle. The protocol precludes inherent trust in any single node’s local AI inference execution, including that of the incumbent leader. Instead, the trust anchor remains the cryptographic quorum of the underlying blockchain. The predictive metrics are safely leveraged because they are derived from the globally consistent, immutable latency history D recorded on-chain. This structural determinism establishes a closed-loop verification process, empowering any honest replica to independently audit and mathematically constrain the leader’s behavior without relying on the integrity of the AI model itself.

Building upon this foundational trust model and leveraging node-specific latency forecasts derived in Section 3.1, we propose an intelligent leader election strategy designed to alleviate single-node bottlenecks and augment consensus efficiency. Unlike conventional BFT protocols, which typically employ a round-robin rotation or a static leader regime to maximize throughput, our approach addresses their inherent limitations. Specifically, the former suffers from periodic performance degradation due to the indiscriminate selection of high-entropy stragglers, while the latter remains agnostic to dynamic nodal performance heterogeneity. Consequently, we introduce an adaptive leader selection strategy that designates the node with the lowest robust ranking metric $R_{i}$—corresponding to the minimal predicted state uncertainty—as the proposer for the subsequent view.

To mitigate the computational and communication overhead associated with rapid leader flapping, we introduce an evaluation window mechanism. The system executes re-election logic strictly at fixed intervals of *W* views. Within this window, the incumbent leader’s tenure is locked to maintain protocol stability. A leadership transition is triggered at the window boundary only when the predictive model indicates a substantial performance divergence between the incumbent and candidate nodes. This hysteresis mechanism acts as a damping factor, ensuring that leadership changes are driven by sustained performance shifts rather than transient stochastic fluctuations.

Formally, let *ℓ* denote the incumbent leader and $j^{*}={\arg\min}_{i\in N}R_{i}\left(t\right)$ be the candidate node exhibiting the optimal predicted performance. A leadership handover to $j^{*}$ is triggered when the relative performance gain exceeds a predefined hysteresis threshold δ:

(5) $$\frac{R_{\ell }\left(t\right)-R_{j^{*}}\left(t\right)}{R_{\ell }\left(t\right)}>\delta$$

where δ is empirically set to 0.1, implying that a leadership change is warranted only when the challenger offers at least a 10% reduction in expected latency. Otherwise, *ℓ* retains its role ($\ell_{t+1}\leftarrow \ell$) to preserve system momentum and avoid overhead from negligible gains.

To guarantee view consistency and deterministic verification among all honest replicas, we implement a piggyback-and-audit mechanism. To synchronize data, the incumbent leader embeds the observed latency vector of the current view, denoted as ${\left\{L_{j}\left(t\right)\right\}}_{j=1}^{N}$, along with the proposed ranking metrics, into the proposal block header.  Upon committing the block, replicas first append the latency vector to their local unified history D, ensuring that all nodes maintain an identical input sequence for the TimesNet model. Subsequently, rather than enforcing rigid bit-level determinism, replicas execute an optimistic acceptance with auditing strategy. Replicas unconditionally adopt the leader’s broadcast metric (${\hat{L}}_{\mathrm{leader}}$) as the reference value to determine the next view’s leader, thereby prioritizing consensus liveness. Simultaneously, for security monitoring, each node feeds the synchronized history D into its local TimesNet instance. The deviation between the broadcast metric and the local prediction is calculated; discrepancies exceeding a predefined anomaly detection threshold ϵ are logged as evidence for future reputation slashing. This mechanism ensures global state synchronization and eliminates the risk of consensus forks.

To guarantee system robustness against potential prediction deviations or adversarial manipulation, the piggyback-and-audit mechanism operates strictly within the safety and liveness boundaries of the underlying BFT protocol. Given that the input history D is rendered immutable post-commitment, the election logic becomes inherently tamper-proof. The formal theoretical analysis regarding how this deterministic fallback automatically purges malicious leaders without compromising system safety is detailed in Section 3.6.

Furthermore, a hybrid switching mechanism is implemented to guarantee system stability during initialization or periods of extreme volatility. Given that the TimesNet model requires a historical sequence of length *k* to accurately capture temporal dependencies, its predictive efficacy is inherently constrained during the initial views. To address this, we define a warm-up window $W_{\mathrm{warmup}}$, set to 50 views in our experiments, ensuring the model captures at least 5 complete network cycles. During the interval $t<W_{\mathrm{warmup}}$, the protocol operates in a cold start mode, functioning passively to accumulate latency data while defaulting to the standard round-robin rotation and static timeout configuration ($\Delta_{\mathrm{static}}$). The AI-driven optimization is activated only when $t\geq W_{\mathrm{warmup}}$. Additionally, to counteract the risk of concept drift, a runtime safety breaker is introduced. If the real-time rolling loss exceeds a predefined safety threshold $\delta_{\mathrm{safe}}$ for ν consecutive views, the system temporarily reverts to the baseline strategy. The stringent system degradation bounds provided by this breaker are further evaluated in Section 3.6.

### 3.3. Adaptive Regulation of Liveness Parameters via Entropy Boundaries

Complementing the entropy-minimizing leader selection strategy, the dynamic calibration of timeout parameters and view tenure determines consensus throughput and liveness. To this end, we leverage TimesNet predictions to regulate protocol timing, balancing system responsiveness with stability through entropy-driven boundary control.

In partially synchronous BFT protocols, critical parameters—such as the proposal voting timeout Δ and the view-change delay—are typically configured as static values. However, this rigid configuration fails to accommodate the time-varying nature of network latency, often resulting in unnecessary view changes or prolonged consensus delays. To address this, we formulate a mechanism to dynamically calibrate the timeout threshold $\Delta_{t}$ based on the incumbent leader’s predicted conditional mean $R_{\ell }\left(t\right)$. Serving as a low-entropy baseline for the network state, this metric facilitates the mitigation of tail entropy risks through dynamic smoothing rather than unstable high-quantile estimation. Inspired by the classic RTT estimation mechanism in TCP protocols [36], we employ an Exponential Moving Average (EMA) strategy to smooth timeout updates. From a signal processing perspective, this EMA acts as a low-pass filter, mitigating oscillatory instability induced by transient, high-frequency stochastic jitter. The adaptive timeout update rule is derived as follows:

(6) $$\Delta_{\mathrm{raw}}=\alpha \cdot R_{\ell }\left(t\right)+\left(1-\alpha \right)\cdot \Delta_{t}$$

where α∈(0,1) represents the smoothing factor, balancing responsiveness to the prediction $R_{\ell }\left(t\right)$ against historical inertia. We adopt α=0.125, aligning with the standard practice in TCP’s RTT estimation to mitigate the impact of transient network jitter. This formulation dictates that if the predicted latency $R_{\ell }\left(t\right)$ drops significantly, signifying a low-entropy state, the system tightens the timeout to expedite consensus; conversely, if high-entropy congestion is forecast, the timeout is relaxed to prevent premature view changes. To ensure systemic stability, the final threshold is constrained within a safe operating range $\left[\Delta_{\min},\Delta_{\max}\right]$. Furthermore, to suppress high-frequency oscillations, we restrict timeout adjustments to periodic calibration intervals of $W_{c}$ views. The finalized periodic update logic is formalized as follows:

(7) $$\Delta_{t+1}=\;\left\{\begin{array}{cc} \min\left(\max\left(\Delta_{\mathrm{raw}},\Delta_{\min}\right),\Delta_{\max}\right) & \mathrm{if}\;t\;\mathrm{mod}\;W_{c}=0\\ \Delta_{t} & \mathrm{otherwise} \end{array}\right.$$

where the conditional execution ensures that parameters remain static between calibration intervals. To ensure responsiveness during high-entropy anomalies, an exceptional timeout recalibration is triggered immediately upon any proactive leader rotation (Section 3.2), overriding the standard calibration interval $W_{c}$ defined in Equation (7).

To balance the trade-off between the high switching overhead of rigid rotation and the liveness risks inherent in static tenure, we propose a dynamic tenure modulation mechanism. Focusing on the leader’s intrinsic entropy stability, we enforce a self-degradation check to prevent intrinsic performance deterioration, where an incumbent node degrades significantly against its own historical baseline yet avoids immediate replacement by peer comparison. A preemptive view change is triggered if the predicted degradation—manifesting as temporal entropy divergence—exceeds a threshold ζ:

(8) $$\frac{R_{\ell }\left(t+1\right)-{\bar{R}}_{\ell }\left(t\right)}{{\bar{R}}_{\ell }\left(t\right)}>\zeta$$

where $R_{\ell }\left(t+1\right)$ denotes the robust latency forecast for the next view, and ${\bar{R}}_{\ell }\left(t\right)$ represents the EMA of the leader’s metric over its current tenure. Consequently, if the relative self-degradation exceeds ζ (empirically set to 0.15, slightly higher than the election threshold δ to prevent hysteresis conflicts), the tenure is terminated immediately, overriding the standard evaluation window to preserve consensus liveness.

These mechanisms are integrated within the protocol Finite State Machine (FSM). The prediction module is invoked at the conclusion of each view to update $\Delta_{t+1}$ and assess leader candidates. Crucially, this modulation is restricted to liveness parameters to ensure that core safety invariants remain preserved. The combined constraints of smoothing, safety bounds, and the cooling-off window act as a multi-stage damping system that prevents control loop instability. Regarding notation, we explicitly align the temporal step *t* in our time-series formulation with the consensus view index. Therefore, in the protocol pseudocode provided in Appendix A, we use the notation *v* and $\ell_{v}$ interchangeably with *t* and $\ell_{t}$ to emphasize the view-based execution flow.

### 3.4. Theoretical Analysis of System Overhead and Complexity

To rigorously evaluate the feasibility of deploying TimesNet-BFT in resource-constrained environments, we analyze the asymptotic bounds of the computational, spatial, and temporal overhead introduced by the deep temporal modeling component.

Regarding the asymptotic time complexity of inference, the TimesNet model processes the latency history of *N* nodes at the end of each view. The inference operation comprises an FFT for frequency-domain analysis and 2D convolutional feature extraction. Given a historical time window of length *k*, the FFT operation incurs a complexity of O(klogk), while the subsequent 2D convolution with *M* channels involves $O(M\cdot k\cdot c^{2})$ operations. Consequently, the total inference time complexity for the entire cluster scales as $O(N\cdot \left(k\log k+M\cdot k\cdot c^{2}\right))$. Since the hyperparameters k,M, and *c* are small constants relative to the network size, the overall complexity exhibits strictly linear growth. This aligns perfectly with the linear communication complexity of the underlying HotStuff protocol, ensuring that the predictive module does not become a computational bottleneck.

In terms of spatial overhead, the memory footprint per node consists of the history state buffer and the static model weights. The history buffer requires storing a sliding window matrix of size N×k. With N=32 and k=60 using 32-bit floating-point precision, this buffer occupies negligible memory (less than 10 KB). Since the model parameter count |Θ| is invariant to the network scale, the total space complexity is bounded by O(N·k+|Θ|), confirming the framework’s suitability for deployment on resource-constrained edge nodes.

Furthermore, to mitigate potential latency inflation on the consensus critical path, the framework employs an asynchronous, pipelined architecture. The inference logic executes in an isolated background thread once per view, predicting the optimal configuration for the subsequent view while the current consensus proceeds. This parallel execution ensures that the main consensus thread simply accesses pre-computed cached variables without blocking. In the worst-case scenario where the inference thread saturates CPU resources ($T_{inference}>T_{network}$), the runtime safety breaker mechanism (Section 3.2) automatically reverts the protocol to the static baseline. Therefore, the worst-case latency overhead imposed by the AI component on the consensus-critical path is effectively eliminated through asynchronous decoupling.

### 3.5. Mathematical Nexus of MSE, Variance Reduction, and System Spectral Entropy

We establish a formal mathematical mapping from time-domain variance to frequency-domain order. The TimesNet model is optimized by minimizing the MSE, effectively defining the optimal predictor as the conditional expectation ${\hat{L}}_{i}\left(t\right)=E\left[L_{i}\left(t\right)|x_{i}\right]$. Mathematically, minimizing MSE is equivalent to minimizing the variance of the prediction residuals, denoted as $\mathrm{Var}(e_{i})$, where $e_{i}\left(t\right)=L_{i}\left(t\right)-{\hat{L}}_{i}\left(t\right)$. This variance reduction process acts as a spectral filter, suppressing high-frequency aleatoric noise while isolating the structured, multi-periodic signal inherent in the network latency dynamics.

According to Parseval’s Theorem, the total energy of the error signal in the time domain—represented by its variance—is proportional to the integral of its Power Spectral Density (PSD) in the frequency domain. A high MSE implies high variance, which corresponds to a broad-band “white noise” spectrum characterized by a uniform probability distribution P(f,t). It is a fundamental information-theoretic property that a uniform distribution maximizes Shannon entropy, a state we define as entropic chaos.

Consequently, by actively minimizing MSE, TimesNet suppresses this broadband noise floor. This optimization forces the PSD P(f,t) to concentrate in narrow frequency bins, resulting in distinct peaks at the dominant harmonic periods. Based on the formal definition of Spectral Entropy:

(9) $$H\left(t\right)=-\sum_{f}P\left(f,t\right)\log_{2}P\left(f,t\right)$$

This spectral concentration drives H(t) toward its theoretical lower bound. Thus, the local optimization of the regression error implies the global minimization of the BFT system’s spectral entropy, facilitating a phase transition from stochastic turbulence to ordered stability.

### 3.6. Security Analysis and System Degradation Boundaries

Integrating machine learning into the consensus critical path introduces novel attack vectors, specifically out-of-distribution (OOD) extrapolation errors and adversarial manipulation. To rigorously mitigate these vulnerabilities, we formalize the system degradation bounds under both stochastic distribution shifts and Byzantine threat models.

Fundamentally, the TimesNet-BFT framework ensures safety by adhering to a strict decoupling axiom: consensus validity is separated from local model inference. The AI predictions exclusively modulate liveness parameters (leader selection and timeout), while the fundamental voting rules and quorum intersection (2f+1) remain invariant. To formalize this decoupling axiom, we provide a rigorous proof demonstrating that the system’s safety properties are invariant to any epistemic uncertainty or adversarial manipulation introduced by the deep temporal model.

**Theorem 1**  (Safety Independence under Epistemic Uncertainty). *Let E denote an arbitrary prediction error matrix produced by the TimesNet inference module $f_{\theta }$, encompassing both out-of-distribution hallucinations and targeted Byzantine falsifications. The safety property of TimesNet-BFT—defined as no two honest replicas committing conflicting blocks at the same view v—is strictly independent of E.*

**Proof.**  We proceed by contradiction, leveraging the quorum intersection properties of the underlying protocol. Assume, for the sake of contradiction, that the system violates safety due to an arbitrary predictive error E, resulting in two honest replicas committing conflicting blocks *B* and $B^{\prime }$ at the same view *v*.

Dictated by the deterministic protocol definition, the commitment of any block necessitates a valid Quorum Certificate (QC) comprising at least 2f+1 valid cryptographic signatures from the replica set N (where |N|=3f+1). Let Q and $Q^{\prime }$ be the sets of replicas that signed the QCs for *B* and $B^{\prime }$, respectively. Thus, we have |Q|≥2f+1 and $|Q^{\prime }|\geq 2f+1$. The intersection of these two quorums is mathematically bounded by:

(10) $$|Q\cap Q^{\prime }\left|=|Q|+\right|Q^{\prime }|-|Q\cup Q^{\prime }|\geq \left(2f+1\right)+\left(2f+1\right)-\left(3f+1\right)=f+1$$

Given that the system operates under the threshold assumption of at most *f* Byzantine faults, the intersection $Q\cap Q^{\prime }$ must necessarily contain at least one honest replica. However, a fundamental invariant governing honest replicas dictates that they will never cryptographically sign two conflicting proposals for the exact same view *v*. This directly contradicts the assumption that both *B* and $B^{\prime }$ obtained valid QCs.

Crucially, because the optimistic acceptance of a latency metric bypasses consensus state validation, the TimesNet output ${\hat{L}}_{i}\left(t\right)$ maps exclusively to the liveness parameters: the view timeout boundary $\Delta_{v}$ and the leader index $\ell_{v}$. Consequently, the state verification logic and the digital signature generation remain strictly isolated from $f_{\theta }$. Therefore, regardless of how E perturbs $\Delta_{v}$ or $\ell_{v}$, the adverse impact is strictly confined to system liveness. The prediction error E inherently possesses no mechanism to forge the cryptographic signatures of honest replicas required to satisfy the 2f+1 threshold. Thus, the safety property holds strictly independent of E.    □

Regarding specific ML-driven Byzantine vectors, such as model poisoning and latency spoofing, the architecture provides deterministic mitigation. Model poisoning is thwarted by the lazy recalibration strategy, which keeps model weights static during the consensus critical path, preventing real-time adversarial gradient injection. Furthermore, if a Byzantine node attempts a denial-of-service attack by spoofing low latency to hijack leadership, the piggyback-and-audit mechanism ensures that the resulting latency spike during the proposal phase triggers the deterministic view-change timeout. The transient liveness delay is strictly upper-bounded by $\Delta_{max}$, ensuring the automated purging of malicious leaders.

Finally, under zero-shot generalization scenarios involving severe covariate shifts or concept drift, the system’s performance degradation is strictly bounded. Let $E_{OOD}$ represent the maximum prediction error induced by OOD latency patterns. The runtime safety breaker monitors the real-time rolling loss; if the distribution shift drives the loss beyond a safety threshold, the system immediately severs the AI control loop and reverts to the Round-Robin baseline. To prevent oscillation between modes during boundary conditions, a hysteresis mechanism is employed (i.e., the system reactivates AI control only when the loss drops significantly below the threshold for a sustained period). Thus, the worst-case system throughput $\Phi_{worst}$ and latency $\tau_{worst}$ are mathematically bounded by the performance of the standard information-agnostic BFT protocol, guaranteeing that the thermodynamic efficiency of TimesNet-BFT never decays below the theoretical baseline.

## 4. Experimental Evaluation

This section empirically validates the proposed TimesNet-BFT framework, specifically testing the hypothesis that minimizing epistemic uncertainty regarding network states directly correlates with enhanced consensus stability. The experimental protocol is stratified into two primary dimensions: Information Extraction Fidelity, which assesses the latency forecasting module’s capacity to decouple valid periodicity from stochastic latency noise against diverse baselines; and Entropy-constrained Consensus Efficiency, which quantifies the resultant improvements in system throughput and latency within a controlled high-entropy simulation environment. All computational experiments were conducted on a high-performance workstation equipped with an Intel Core i9 processor, an NVIDIA RTX 4090 GPU, and 256 GB of RAM. The framework was implemented using PyTorch 2.1 on Ubuntu 22.04 to ensure reproducibility, with both the state-estimating inference engine and the consensus logic executed on a unified platform.

### 4.1. Datasets and Experimental Configuration: Modeling High-Entropy Simulation Environments

To rigorously evaluate the thermodynamic boundaries of the consensus protocol under extreme conditions, we constructed a controlled high-entropy simulation environment rather than relying solely on static historical traces. This approach allows us to systematically inject “Entropy Singularities” to stress-test the system’s phase stability, ensuring reproducible and verifiable entropy dynamics analysis.

We established a distributed Peer-to-Peer network topology comprising N=32 geographically dispersed nodes. This cluster configuration serves as a stochastic testbed representative of production-grade consortium environments. To rigorously challenge the information extraction capabilities, the end-to-end latency $T_{i,j}\left(t\right)$ is modeled as a Stochastic Superposition Process, encompassing multiple entropy sources:

(11) $$T_{i,j}\left(t\right)=T_{prop}+T_{super}\left(t\right)+T_{congestion}\left(t\right)\;$$

where $T_{prop}$ represents the base propagation delay, determined by the simulated geographic distance between nodes and fixed within the range [30ms,80ms] to simulate physical link constraints. The second component, $T_{super}\left(t\right)$, introduces structured temporal entropy. We inject a base wave ($T_{base}=20$ views) to represent tidal effects, superimposed with a high-frequency jitter ($T_{rand}\in \left[4,8\right]$ views) to simulate rapid fluctuations. Distinct phase shifts $\phi_{i}$ are assigned to each node to simulate network asynchrony. Finally, $T_{congestion}\left(t\right)$ accounts for aleatoric burst noise arising from unpredictable congestion. We employ a Poisson process to trigger sporadic packet loss; upon activation, specific request latencies spike to a uniformly distributed range of [150ms,350ms], generating a realistic long-tail distribution. Additionally, to simulate a sudden phase transition induced by catastrophic failure, we introduce a deterministic Partial Network Failure event. In this scenario, 25% of the network nodes (Nodes 0–7) experience a catastrophic latency surge to 600 ms, representing a localized subnet paralysis, as visualized later in the spectral analysis.

Using this high-fidelity modeling approach, we generated time-series data spanning 1000 consensus views. The initial 80% (800 views) was employed for supervised training of TimesNet, enabling the model to capture the manifold of valid network states. Notably, since the model is trained on data from all N=32 nodes, the effective training corpus comprises 25,600 node-view samples, providing sufficient data density for the deep learning model. To further mitigate potential data scarcity and accelerate convergence, we adopted a transfer learning strategy, initializing the model with weights pre-trained on standard time-series benchmarks before fine-tuning on our consensus logs. To ensure numerical stability and model convergence during optimization, we applied weight decay ($1\times 10^{-4}$) to penalize structural entropy and gradient clipping (1.0) to curb explosive updates. The remaining 20% (200 views) was reserved for online inference and consensus performance validation. Consistent with the asynchronous design detailed in Section 3.2, the TimesNet inference engine operates in a non-blocking background thread, thus imposing minimal blocking latency on the consensus critical path. The experimental timeline is divided into two phases: an offline pre-training phase (t<800), during which the model learns from historical logs under a standard round-robin policy, and an online evaluation phase (t≥800), during which the pre-trained TimesNet model assumes active control over leader selection.

To rigorously assess the model’s generalization capabilities within non-stationary high-entropy environments, we employed a Trace-Driven Simulation approach based on the VeReMi (Vehicular Reference Misbehavior) dataset [37] as a zero-shot testing benchmark. This dataset serves as a recognized standard for IoV security research, providing realistic Vehicle-to-Vehicle message logs characterized by stochastic channel fading and malicious behavior injection. Specifically, we extracted a continuous sequence of message transmission latencies (calculated as $\Delta t=T_{rcv}-T_{send}$ from the message logs) comprising 5000 timestamps from the position forgery scenario. This specific subset exhibits severe jitter and long-tail latency distributions—structural characteristics strictly analogous to the network instability induced by consensus attacks. To ensure numerical stability during the inference phase, the raw latency values underwent Z-Score standardization. This dataset was strictly excluded from the training corpus to verify the transfer robustness of TimesNet against unseen entropy distributions.

To precisely isolate the impact of network latency on consensus performance, a Discrete-Event Simulator (DES) was developed to faithfully implement the core FSM of the standard HotStuff protocol. The simulator strictly adheres to the canonical three-phase commit workflow (encompassing the Prepare, Pre-commit, and Commit stages), while implementing a Quorum mechanism requiring 2f+1 votes. Furthermore, to focus the evaluation exclusively on transport-layer entropy, cryptographic primitives are modeled as deterministic constants fixed at 2 ms. Thus, any fluctuations in total consensus latency are attributed solely to network transmission variables.

In our comparative evaluation, two distinct consensus strategies were instantiated to benchmark performance. The baseline strategy adopts the standard rotating-leader HotStuff protocol with a fixed view timeout threshold of Δ=200ms. This aggressive configuration models latency-critical environments and serves as a boundary test, evaluating protocol resilience with a static, information-agnostic boundary just above the physical propagation delay. It adheres to a deterministic round-robin policy where leadership rotates sequentially among replicas (L=(vmodN)+1), regardless of the incumbent’s performance. In contrast, the TimesNet-BFT framework integrates the TimesNet inference engine within the DES. At the conclusion of each view, the system executes parallel latency prediction to derive the robust ranking metric $R_{i}$, and adaptively modulates the timeout threshold $\Delta_{t+1}$ based on the entropy-driven logic defined in Section 3.3.

To ensure a rigorous comparison, we executed 200 consensus views for both schemes under identical network latency trajectories governed by fixed random seeds. Note that while quantitative metrics are derived from the full 200-view dataset, the temporal visualizations presented in Section 4.3 focus on the initial 125 views to explicitly demonstrate the system’s transient response to injected anomalies with higher resolution. Crucially, to ensure the physical interpretability of evaluation metrics, all predicted values were inverse-transformed to the original millisecond scale prior to the calculation of Mean Absolute Percentage Error (MAPE).

### 4.2. Fidelity of Latency Information Extraction

This section conducts a systematic comparative analysis to evaluate the model’s capacity to minimize epistemic uncertainty across two distinct noise regimes. First, we assess the information extraction fidelity on the multi-node simulation dataset (generated in Section 4.1) to establish a baseline for reconstructing valid signals from stochastic noise. Subsequently, we evaluate transfer robustness via a zero-shot inference regime using VeReMi-driven traces to test the model’s generalization capabilities across heterogeneous entropy domains.

To comprehensively assess the fidelity of state estimation, we benchmark our approach against three representative paradigms spanning statistical and deep learning domains: ARIMA (Auto-Regressive Integrated Moving Average) [38] as a classical method assuming linear stationarity, RBFNN (Radial Basis Function Neural Network) [39] representing shallow non-linear approximation, and LSTM [40] as a deep architecture capturing temporal dependencies via recurrent memory. To ensure a fair comparison, all models were trained on the identical simulation subset, comprising the initial 800 views. Crucially, hyperparameters were carefully optimized via grid search to ensure each model operates at its peak capacity for pattern recognition. The specific hyperparameter configurations are detailed in Table 1.

To quantitatively evaluate the prediction accuracy, the single-step prediction fidelity across both the simulation test set (the final 200 views) and the real-world traces is presented in Table 2. In the simulated environment, the results demonstrate that TimesNet achieves superior performance, recording a minimal residual uncertainty with a MAPE of 4.75%. Relative to the strong LSTM baseline (6.09%), TimesNet reduces the predictive residual entropy by approximately 37%. Furthermore, under the rigorous zero-shot evaluation regime on the VeReMi-driven logs, while the complex real-world data induces performance drops across all models due to severe distribution shift, TimesNet maintains competitive precision (MAPE of 23.72%). It demonstrates structural robustness comparable to the heavy recurrent architecture of LSTM (23.80%) and significantly outperforms statistical models like RBFNN (35.89%) and ARIMA (29.19%), which struggle with non-linear volatility.

**Figure 2:**
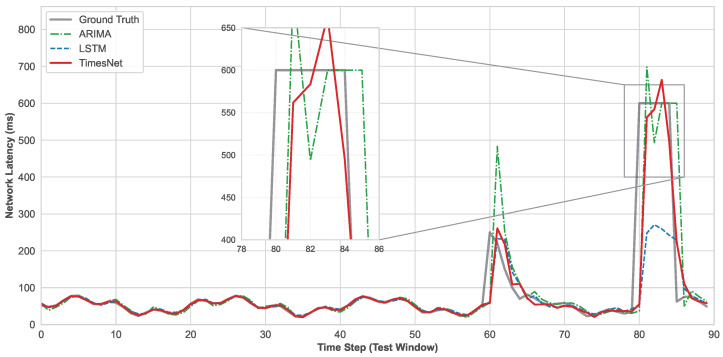
Latency forecasting performance. Comparative analysis of a representative node shows TimesNet achieves superior convergence with ground truth, accurately tracking high-entropy singularities where baselines exhibit significant phase lag or instability.

To elucidate the temporal dynamics underlying this quantitative superiority, Figure 2 plots the single-step prediction fitting curves for the simulated environment. The overall trend indicates that the TimesNet trajectory exhibits tight convergence with the ground truth, accurately tracking multi-scale periodic fluctuations. In contrast, the ARIMA curve exhibits erratic fluctuations and significant overshoot, failing to model the complex non-linear volatility.

Crucially, the magnified inset in Figure 2 highlights the models’ responses to a critical network failure event, characterized by a high-entropy singularity manifesting as a catastrophic latency surge to 600 ms. Recurrent architectures like LSTMs exhibit a pronounced informational phase lag and severe amplitude attenuation, with the predicted value failing to exceed 260 ms, thereby dangerously underestimating the system risk. By systematically decomposing complex latency time series into distinct periodic components via FFT, TimesNet accurately distinguishes between structured dual-frequency fluctuations and sporadic high-entropy anomalies. This enables the model to promptly capture the phase transition and accurately predict the recovery trajectory without the hysteresis inherent in RNNs. Ultimately, this robust time-frequency representation enables TimesNet to effectively capture latency dynamics across heterogeneous consensus environments.

### 4.3. Entropy-Constrained Consensus Efficiency Evaluation

This section provides a quantitative evaluation of the thermodynamic efficiency enhancements realized by TimesNet-BFT. To rigorously assess system throughput, transaction confirmation latency, and phase stability (view change frequency), the experimental evaluation is structured around two distinct entropy regimes: Nominal and Adverse.

The Nominal Scenario emulates a stable operating environment characterized by minor stochastic fluctuations around the baseline network latency. This scenario serves as a control baseline to verify that the optimization framework introduces negligible computational entropy under normal conditions. Conversely, the Adverse Scenario is designed to stress-test system resilience through two specific classes of anomalies. Initially, spanning Views 50–70, the system is subjected to network jitter, where high-variance latency fluctuations are simulated via the systematic injection of multi-scale stochastic noise to mimic channel instability. Subsequently, commencing at View 80, a partial network failure is triggered, representing a structural topology breakdown. In this scenario, a distinct subset of nodes (25% of the cluster) experiences a catastrophic latency surge to 600 ms, far exceeding the 200 ms timeout boundary. This comparative analysis evaluates the information-agnostic baseline protocol against the entropy-aware TimesNet-BFT.

**Figure 3:**
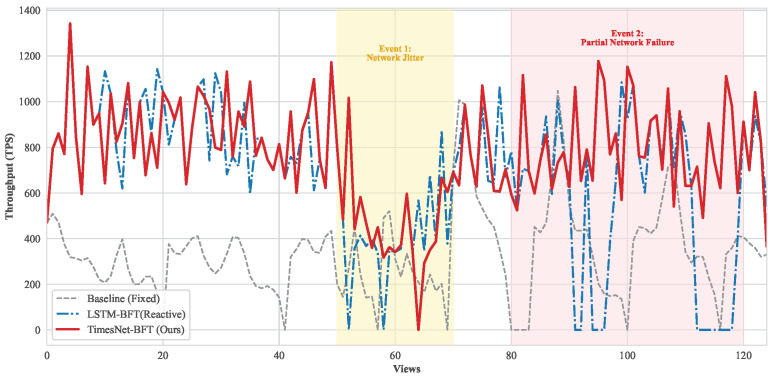
Temporal evolution of system throughput under stochastic network conditions. The comparative analysis highlights the resilience of TimesNet-BFT during the network jitter phase (Event 1) and its capability to completely bypass compromised nodes during the partial network failure phase (Event 2), whereas the LSTM-BFT suffers from periodic collapses.

Table 3 summarizes the key performance metrics. In the nominal scenario, TimesNet-BFT distinguishes itself by perceiving the inherent stability of the network, allowing the protocol to dynamically tighten the timeout boundary. By replacing the conservative static baseline with this optimized low-entropy bound, the system effectively recovers idle waiting time that would otherwise be dissipated as computational waste in fixed-parameter settings. This transition from static to proactive adjustment directly translates into a noticeable leap in system throughput and a corresponding reduction in confirmation latency. While LSTM-based approaches (visualized in Figure 3) can also adapt, they suffer from informational phase lag during anomalies. In contrast, TimesNet-BFT ensures superior responsiveness, eliminating the hysteresis observed in recurrent architectures.

Figure 3 illustrates the real-time throughput trajectory, explicitly demonstrating the divergence in entropy management strategies. During the network jitter phase (Event 1, yellow zone), significant performance divergences are observed. As global network entropy rises due to channel instability, the static strategy exhibits erratic oscillations, frequently dropping due to timeout violations. In contrast, TimesNet-BFT adapts its timeout threshold, maintaining a robust throughput baseline (461 TPS) significantly higher than the baseline (236 TPS).

Subsequently, the partial network failure event serves as a critical differentiator. In this phase, 25% of the nodes experience severe latency degradation. The LSTM-BFT curve (blue dashed) exhibits a characteristic periodic collapse, dropping to zero TPS whenever the rotation selects a compromised node as leader. In sharp contrast, TimesNet-BFT maintains a consistently high throughput trajectory. By leveraging deep temporal feature extraction, the system proactively identifies and excludes these high-entropy stragglers from the leadership pool, effectively neutralizing the impact of the partial network paralysis.

**Figure 4:**
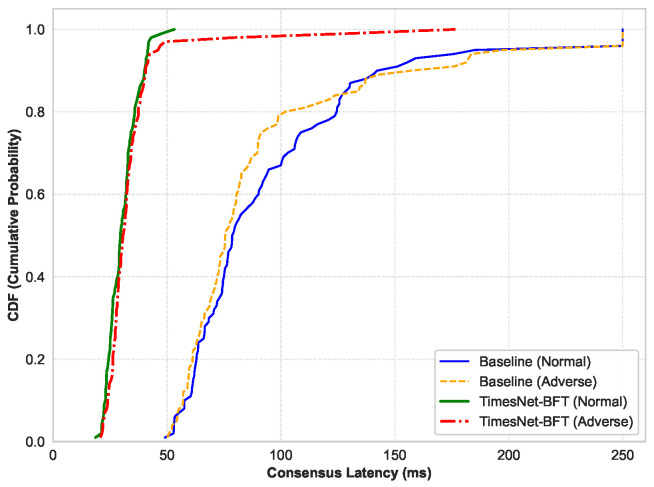
CDF of confirmation latency. TimesNet-BFT shows a steep ascent, indicating low latency variance, whereas the Baseline exhibits a significant long-tail distribution under adverse conditions, validating the mitigation of entropy-induced idle waits.

To provide a granular dissection of system latency characteristics, Figure 4 delineates the Cumulative Distribution Function (CDF) of transaction confirmation latencies. Beyond the statistical variance, where the optimized scheme standard deviation of 14.28 ms is significantly lower than the Baseline value of 46.81 ms, the CDF curves unveil fundamental disparities in tail entropy management.

As illustrated in Figure 4, the optimization schemes depicted by the green and red curves exhibit an extremely steep vertical ascent, reaching saturation approaching the 50 ms mark. This morphology indicates that the vast majority of transactions are confirmed within a highly narrow latency interval, signifying a collapse of state uncertainty. Notably, the TimesNet-BFT curve under adverse conditions maintains high fidelity to its normal counterpart, manifesting negligible performance degradation even under stress.

In sharp contrast, the baseline scheme under adverse conditions (orange dashed line) demonstrates a significant rightward shift and a distinct long-tail distribution extending towards the 250 ms mark. This empirical evidence corroborates the structural inefficiency of static timeout policies, in which a substantial portion of transactions must endure entropy-induced idle waits caused by timeout expirations prior to recovery. TimesNet-BFT effectively mitigates this long-tail bottleneck via adaptive timeout calibration, ensuring robust liveness and significantly reducing the worst-case latency compared to the static baseline.

**Figure 5:**
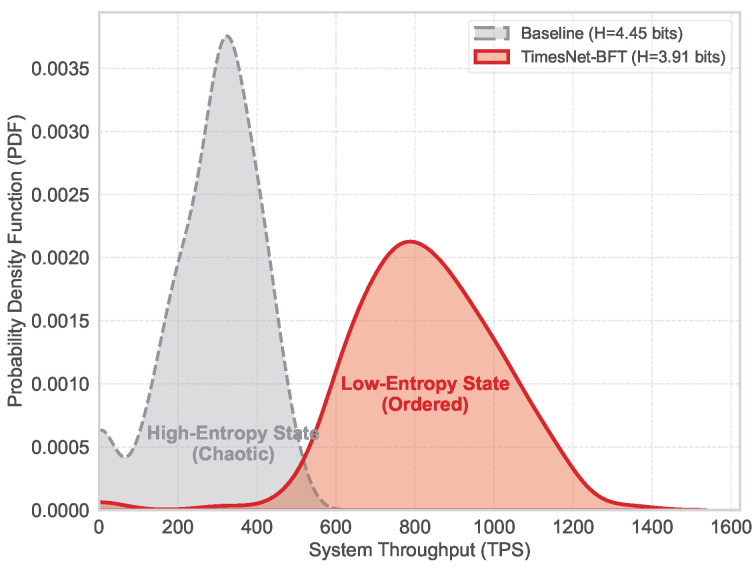
Statistical probability density of system throughput. The Baseline (gray) shows a high-entropy dispersed distribution, while TimesNet-BFT (red) demonstrates a low-entropy concentration, signifying minimized performance uncertainty.

This stability is verified via spectral entropy analysis in Figure 5, where TimesNet-BFT exhibits a concentrated leptokurtic distribution (H≈3.91 bits) compared to the dispersed Baseline (H≈4.45 bits).

Regarding resource utilization efficiency, the analysis reveals a fundamental shift in consensus participation strategy. The baseline Round-Robin scheme, while theoretically fair, suffers from inefficient resource allocation by indiscriminately forcing straggler nodes to lead, thereby impeding the global consensus speed. In contrast, the optimized framework adopts a performance-oriented meritocracy. By concentrating leadership responsibilities on TimesNet-predicted high-performance nodes, the system maximizes the utilization of superior computational and network resources. While this introduces a trade-off by reducing the participation rate of edge nodes, it effectively circumvents the “straggler bottleneck”. Crucially, unlike static centralization, this meritocracy is dynamic—leadership rotates based on real-time entropic states rather than fixed identities, ensuring that the system operates near its optimal throughput capacity without permanently excluding recovering nodes.

**Figure 6:**
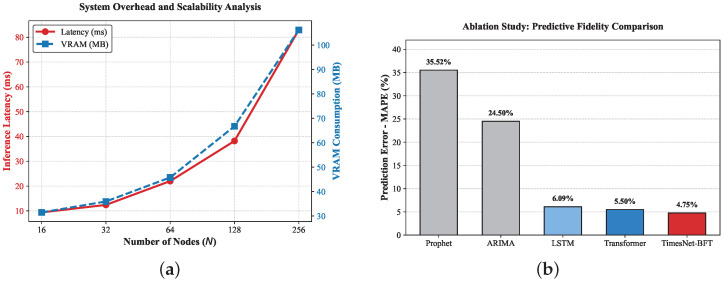
Comprehensive performance evaluation. (**a**) The proposed framework maintains a strict O(N) linear latency scaling (red) with minimal VRAM footprint (blue), confirming feasibility for edge deployment. (**b**) The ablation study demonstrates that TimesNet-BFT (MAPE 4.75%) significantly outperforms 1D attentive models (Transformer) and statistical baselines by effectively capturing multi-scale periodicities.

Finally, regarding computational scalability, we experimentally validated the theoretical bounds discussed in Section 3.4. As shown in Figure 6a, the inference latency scales linearly (O(N)) from 9.39 ms (N=16) to 82.96 ms (N=256). Crucially, the VRAM consumption remains negligible (<110 MB), confirming that the deep temporal modeling introduces minimal resource overhead and is suitable for deployment on computational edge nodes.

### 4.4. Zero-Shot Generalization to Wide Area Networks

To assess the framework’s robustness against severe distribution shifts, we extended the evaluation to a simulated WAN scenario. Unlike the microsecond jitter in IoV, the WAN environment is characterized by high base latencies ($T_{\mathrm{prop}}\in \left[100,250\right]$ ms) and macroscopic route flapping events.

**Figure 7:**
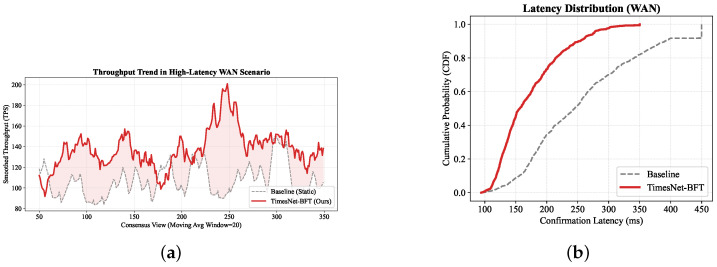
WAN scenario performance. (**a**) TimesNet-BFT maintains higher TPS by actively filtering slow paths. (**b**) The CDF shows a sharp reduction in tail latency compared to the static baseline.

We adopted a rigorous zero-shot protocol: the TimesNet model, trained exclusively on the IoV dataset (Section 4.1), was deployed directly into this WAN environment without any fine-tuning. The comparative results are presented in Table 4 and Figure 7.

As illustrated, TimesNet-BFT demonstrates remarkable transferability. Despite the significant covariate shifts, the model effectively reduces the average latency by 33.2%. Interestingly, the system exhibits a noticeably higher frequency of view changes compared to the baseline. This phenomenon indicates the emergence of a strategy we explicitly define as *aggressive liveness*: rather than passively tolerating the long-tail latency inherent in the static timeout (set to 400 ms), the entropy-aware agent proactively rotates leadership upon detecting route congestion. Crucially, this approach prioritizes temporal determinism over rotation stability to maintain high performance under severe network stress. This trade-off—accepting higher switching costs to secure a lower consensus latency floor—confirms that the multi-periodicity features learned from IoV dynamics are structurally transferable to macroscopic network anomalies.

To rigorously evaluate the control-plane cost and sustainability of this aggressive liveness paradigm, we quantified the supplementary network bandwidth consumption introduced by the AI-driven architecture across two dimensions. First, regarding the payload overhead, embedding the N=32 node latency vector into the proposal block header incurs an extra payload of approximately 128 bytes; relative to a typical 1 MB transaction batch, this represents a mathematically negligible expansion of roughly 0.012%. Second, regarding the rotational overhead induced by the adaptive strategy, which triggers more frequent view changes to circumvent stragglers, the aggregate extra control traffic generated over the evaluation period was strictly constrained to approximately 57 KB. Consequently, given that this mechanism facilitates a verified 33.2% reduction in average consensus latency, the micro-scale increase in control traffic constitutes an exceptionally efficient thermodynamic trade-off, maximizing systemic throughput and responsiveness.

### 4.5. Discussion: Entropy Reduction as a Stability Catalyst

**Figure 8:**
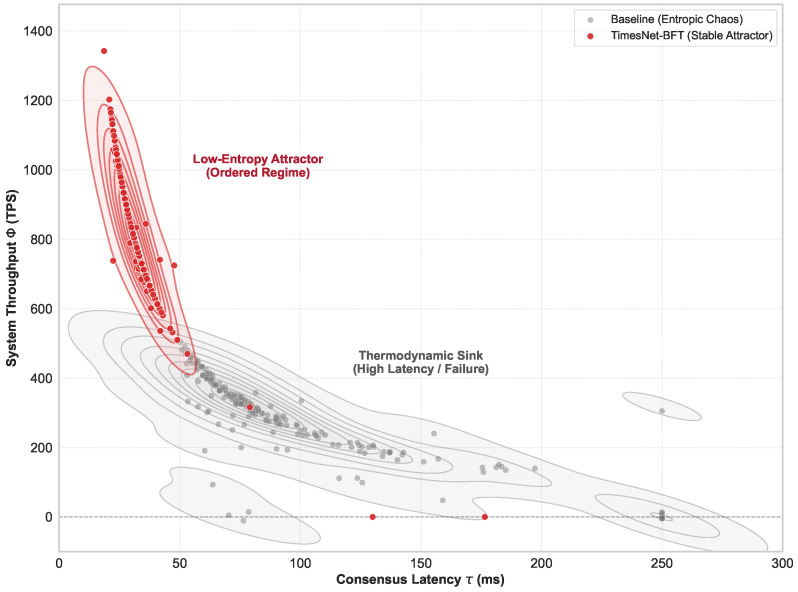
Phase space trajectories of system dynamics. The system state evolution is projected onto the Latency–Throughput plane, where the horizontal dashed line denotes the zero-throughput baseline. TimesNet-BFT converges to a stable attractor (red), maintaining equilibrium, in contrast to the Baseline protocol, which diverges into a high-entropy, chaotic regime (gray).

The experimental results substantiate the fundamental hypothesis grounded in information dynamics: minimizing epistemic uncertainty is a prerequisite for maximizing the stability of distributed systems. To rigorously quantify this stability, we formally define the System Entropy H(t) via Spectral Entropy, which serves as a metric for the structural complexity of the network state. Let P(f,t) be the normalized PSD of the leader’s observed latency sequence. Recalling the formal definition of Spectral Entropy formulated in Section 3.5 (Equation (9)), a lower H(t) indicates a highly ordered state dominated by structured periodicities, thereby minimizing the information entropy of the consensus process. Consequently, as visualized in the Entropy-Throughput phase space (Figure 8), TimesNet-BFT demonstrates convergence toward a stable “Low-Entropy Attractor” in the high-performance quadrant, effectively mitigating the entropic drift characteristic of the baseline protocol.

**Figure 9:**
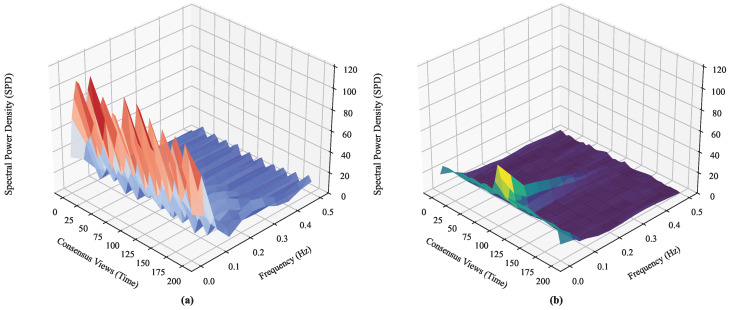
Evolution of spatiotemporal spectral entropy. The colors indicate the magnitude of the spectral entropy. (**a**) The Baseline exhibits high-entropy turbulence dominated by broadband noise. (**b**) TimesNet-BFT effectively suppresses jitter, revealing structured harmonics and facilitating a phase transition from stochastic chaos to ordered periodicity.

This macroscopic stability stems from spectral filtering at the signal level. As visualized in Figure 9, traditional BFT protocols typically operate in a high-entropy turbulent regime characterized by broadband noise. In contrast, TimesNet-BFT functions analogously to a “Maxwell’s Demon” actively filtering stochastic fluctuations to minimize system entropy. By optimizing the metric defined in Equation (9), our framework facilitates a transition of the latency distribution from a disordered stochastic state to an ordered harmonic state. This substantiates that the observed performance gains are not merely engineering optimizations, but the outcome of a systemic phase transition driven by information gain.

Traditional BFT protocols operate under conditions of information opacity, utilizing static parameters that presume a stationary environment. When the network exhibits high entropy—characterized by jitter and node failures—this information asymmetry leads to significant resource dissipation in the form of idle waits and redundant view changes. Our framework conceptually transforms the consensus mechanism into an Information Engine. By leveraging TimesNet to extract structured periodicity from stochastic noise and thereby achieving information gain, the system effectively converts this information into work, specifically by dynamically tightening timeout boundaries and proactively selecting low-latency leaders.

Table 3 quantitatively corroborates this theoretical linkage: the predictive precision directly correlates with the suppression of macroscopic system variance, evidenced by a 92.8% reduction in view change frequency and a 73.3% decrease in latency standard deviation.

## 5. Conclusions

This study proposes TimesNet-BFT, an entropy-aware optimization framework that integrates deep time-series forecasting into the BFT consensus layer to minimize epistemic uncertainty. To address intrinsic stochastic inefficiencies arising from information-agnostic constraints like rigid timeout configurations, the proposed framework leverages the TimesNet architecture to transform consensus from a reactive state to a proactive, low-entropy adaptation posture. Empirical evaluations across both simulated and real-world trace-driven scenarios validate that this uncertainty-minimization approach significantly enhances systemic efficiency. Specifically, it achieves up to a 191.9% increase in throughput and a 73.3% reduction in latency standard deviation, ensuring robust system stability under high-entropy network conditions. Compared to static baselines, TimesNet-BFT achieves a seamless transition during abrupt node failures and maintains optimal operation through entropy-driven parameter modulation, effectively mitigating the resource dissipation typically associated with rigid timeout mechanisms. Moreover, by formally decoupling consensus safety from epistemic uncertainty, the framework introduces a novel aggressive liveness paradigm. This approach trades mathematically negligible control-plane overhead for a 33.2% reduction in average latency in Wide Area Networks, establishing a highly efficient thermodynamic balance.

While this work establishes a robust foundation for information-theoretic consensus design, future research will aim to further enhance the fidelity of state estimation by incorporating multidimensional entropy sources, such as bandwidth fluctuations and nodal computational loads. Additionally, we intend to explore federated learning architectures to enable nodes to collaboratively refine shared models while preserving data privacy. Ultimately, this study demonstrates the significant potential of combining AI with distributed ledger technology, laying a technical cornerstone for the next generation of autonomous and self-organizing consensus architectures.

## Figures and Tables

**Table 1:** Detailed experimental configuration and model hyperparameters.

Component	Category	Key Parameters
ARIMA	Baseline Model	Auto-ARIMA (Adaptive Order Selection)
LSTM	Baseline Model	Window Size: 20, Hidden Units: 50, Layers: 2
RBFNN	Baseline Model	Hidden Layer: 40 Radial Basis Function Units
TimesNet-BFT	Proposed Model	Seq = 60, $d_{model}$ = 64, Top-*k* = 5, Kernels = 6
Consensus Logic	Protocol Settings	Δ=200 ms, δ=0.10, α=0.125
Simulation	Noise Environment	Dual-Freq. (Base + High-Freq. Jitter)

**Table 2:** Prediction accuracy and generalization analysis: simulation vs. real-world-driven env.

Model	Sim. Environment MAPE (%)	Real-World Driven Env. MAPE (%)
ARIMA	24.50	29.19
RBFNN	34.86	35.89
LSTM	6.09	23.80
TimesNet	4.75	23.72

**Table 3:** Consensus performance comparison: Nominal vs. Adverse Scenarios. (TPS: transactions per second; Jitter: injected noise phase; σ: statistical dispersion of latency. The symbols ↑ and ↓ denote the percentage increase and reduction relative to the baseline, respectively).

Test Scenario	Metric	Comparative Scheme		Improvement
		Baseline (Fixed)	TimesNet-BFT	
Nominal	Mean TPS	299.26	873.72	↑191.9%
Nominal	Mean Confirmation Latency (ms)	94.53	29.87	↓68.4%
Adverse	Jitter TPS	236.08	461.39	↑95.4%
Adverse	View Change Freq.	14	1	↓92.8%
Adverse	Latency Std. Dev. (σ)	54.51 ms	14.55 ms	↓73.3%

**Table 4:** Zero-shot performance in the WAN scenario. (The symbols ↑ and ↓ denote the percentage increase and reduction relative to the baseline, respectively).

Metric	Baseline (Static)	TimesNet-BFT	Relative Change
Avg Throughput (TPS)	105.48	136.70	↑ 29.6%
Avg Latency (ms)	257.66	172.15	↓ 33.2%
View Change Freq.	82	196	↑ 139.0% (Adaptive)

## Data Availability

The VeReMi dataset analyzed during the current study is publicly available in the VeReMi repository [37]. The simulation datasets generated and analyzed in the current study are publicly available in our GitHub repository (v1.0), and can be accessed at https://github.com/invinciblehaolong/timesnet-bft-datasets (accessed on 26 January 2026).

## Abbreviations

    The following abbreviations are used in this manuscript:

AI	Artificial Intelligence
ARIMA	Auto-Regressive Integrated Moving Average
BFT	Byzantine Fault Tolerance
CDF	Cumulative Distribution Function
DAG	Directed Acyclic Graph
DES	Discrete-Event Simulator
EMA	Exponential Moving Average
FFT	Fast Fourier Transform
FL	Federated Learning
FSM	Finite State Machine
IoT	Internet of Things
IoV	Internet of Vehicles
LSTM	Long Short-Term Memory
MAPE	Mean Absolute Percentage Error
PBFT	Practical Byzantine Fault Tolerance
RBFNN	Radial Basis Function Neural Network
RL	Reinforcement Learning
RNN	Recurrent Neural Network
TPS	Transactions per Second
UAV	Unmanned Aerial Vehicle
WAN	Wide-Area Network

## Appendix A. Pseudocode of TimesNet-BFT Consensus Loop

**Algorithm A1** TimesNet-driven adaptive BFT Consensus Loop.	
Require: History D, View *v*, Timeout $\Delta_{v}$, Leader $\ell_{v}$	
Ensure: Next View v+1, Leader $\ell_{v+1}$, Timeout $\Delta_{v+1}$	
1: **Phase 1: Normal Consensus Execution**	
2: Node *i* executes HotStuff($v,\ell_{v},\Delta_{v}$)	▹ Standard BFT
3: **if** Block $B_{v}$ is Committed **then**	
4: Extract latency vector $L_{obs}$ from $B_{v}$.Metadata	
5: $D\leftarrow D\cup \{L_{obs}\}$	▹ History sync, Equation (1)
6: **else**	
7: Trigger ViewChange	▹ System Reset due to High Entropy
8: **end if**	
9: **Phase 2: TimesNet Inference**	
10: **if** $\left|D\right|\geq W_{\mathrm{warmup}}$ **then**	
11: **for** each node j∈N **do**	
12: ${\hat{L}}_{j}\leftarrow \mathrm{TimesNet}\left(D{|}_{j},\theta \right)$	▹ Predict, Equation (2)
13: $R_{j}\leftarrow {\hat{L}}_{j}$	▹ Assign Robust Metric
14: **end for**	
15: IsReady←True	
16: **else**	
17: IsReady←False	▹ Insufficient history
18: **end if**	
19: **Phase 3: Dynamic Parameter Modulation**
20: **if not** IsReady **then**	▹ Cold Start Mode
21: $\ell_{v+1}\leftarrow \left(\ell_{v}\;\mathrm{mod}\;N\right)+1$	▹ Fallback: Round-Robin
22: $\Delta_{v+1}\leftarrow \Delta_{\mathrm{static}}$	▹ Fixed Timeout
23: **else**	▹ AI-Driven Mode
24: $j^{*}\leftarrow \arg\min_{j\in N}R_{j}$	
25: $\mathrm{Degrade}\leftarrow \left(R_{\ell_{v}}-\mathrm{EMA}\left(R_{\ell }\right)\right)/\mathrm{EMA}\left(R_{\ell }\right)$	
26: // 1. Leader Selection Logic	
27: **if** $\left(R_{\ell_{v}}-R_{j^{*}}\right)/R_{\ell_{v}}>\delta$ **or** Degrade>ζ **then**	
28: $\ell_{v+1}\leftarrow j^{*}$	▹ Proactive Entropy Reduction, Equation (5)
29: **else**	
30: $\ell_{v+1}\leftarrow \ell_{v}$	▹ Keep Incumbent
31: **end if**	
32: // 2. Timeout Calibration Logic	
33: **if** $v\;\left(\mathrm{mod}\;W_{c}\right)=0\vee \ell_{v+1}\neq \ell_{v}$ **then**	
34: $\Delta_{\mathrm{raw}}\leftarrow \alpha \cdot R_{\ell_{v+1}}+\left(1-\alpha \right)\cdot \Delta_{v}$	▹ Equation (6)
35: $\Delta_{v+1}\leftarrow \mathrm{Clip}\left(\Delta_{\mathrm{raw}},\Delta_{\min},\Delta_{\max}\right)$	▹ Cooling-off
36: **else**	
37: $\Delta_{v+1}\leftarrow \Delta_{v}$	
38: **end if**	
39: **end if**	
40: **return** $v\leftarrow v+1,\ell_{v+1},\Delta_{v+1}$	
